# Physiological and metabolic fluctuations of the diatom *Phaeodactylum tricornutum* under water scarcity

**DOI:** 10.3389/fmicb.2025.1555989

**Published:** 2025-03-19

**Authors:** Ting-Bin Hao, Peng-Yu Lai, Zhan Shu, Ran Liang, Zhi-Yun Chen, Ren-Long Huang, Yang Lu, Adili Alimujiang

**Affiliations:** ^1^School of Stomatology, College of Life Science and Technology, Jinan University, Guangzhou, China; ^2^College of Synthetic Biology, Shanxi University, Taiyuan, China; ^3^Guangzhou Zhixin High School, Ersha Campus, Guangzhou, China

**Keywords:** polyethylene glycol, water stress, lipid, *Phaeodactylum tricornutum*, diatom

## Abstract

Water scarcity is an escalating environmental concern. The model diatom, *Phaeodactylum tricornutum*, holds promise as a potential cell factory for the production of high-value natural compounds. However, its dependence on saline water cultivation restricts its use in areas facing water shortages. Although numerous studies have delved into the metabolic mechanisms of plants under water stress, there is a limited understanding when it comes to microalgae. In our study, we employed polyethylene glycol (PEG) to simulate water scarcity conditions, and assessed a range of parameters to elucidate the metabolic responses of *P. tricornutum*. Water stress induced the generation of reactive oxygen species (ROS), curtailed the photosynthetic growth rate, and amplified lipid content. Our insights shed light on the physiology of *P. tricornutum* when subjected to water stress, setting the stage for potential applications of microalgae biotechnology in regions grappling with water scarcity.

## Highlights

- Water scarcity stress inhibited the photosynthetic growth of microalgae.
- Water stress prompted microalgae to release excessive ROS.
- Under water stress, there was a significant accumulation of TAG.
- Fatty acids underwent *de novo* synthesis under water stress.

## 1 Introduction

Marine diatoms have garnered substantial attention due to their capability to produce a range of commercially valuable products such as polyunsaturated fatty acids, carotenoids, polysaccharides, and proteins. They are viewed as promising candidates for large-scale cultivation (Yang et al., 2020). However, to unlock this potential, it’s imperative to make their mass cultivation economically sustainable by considerably reducing water costs (Gouveia et al., 2016). Water is pivotal for nutrient absorption, determining cell morphology, and facilitating the photosynthetic growth of microalgae (Curien et al., 2016; Yang et al., 2011). Consequently, inland regions distant from the ocean face challenges in large-scale diatom cultivation due to limited water availability, which significantly curtails the commercial prospects of marine diatoms.

Water stress, stemming from water scarcity, increasingly constrains socio-economic development and endangers livelihoods worldwide. The rapidly growing population, economic expansion, and shifting consumption habits has led to severe worldwide water shortages and pollution (Wang et al., 2021). These issues pose significant threats to human health, the environment, and sustainable development (Chowdhary et al., 2020). Projections indicate that by 2050, more than half of the world’s population will live in regions experiencing water stress (Lee et al., 2016). Addressing this scarcity poses a formidable challenge for future endeavors. Water stress acts as a prominent environmental stressor, inducing biochemical, molecular, and physiological alterations that detrimentally affect plant growth and development (Farooq et al., 2012; Rosa et al., 2020). In defense against water stress, plants exhibit a myriad of adaptive strategies, encompassing gene up- or down-regulation, transient spikes in abscisic acid, accumulation of compatible solutes and protective enzymes, enhanced antioxidant concentrations, and the modulation of energy-intensive pathways (Farooq et al., 2012; Basu et al., 2016). However, limited research has been directed toward understanding the physiology and metabolism of diatoms under water stress conditions.

As one of the most extensively studied diatoms, *Phaeodactylum tricornutum* exhibits superior characteristics in comparison to its counterparts. Its adaptability as a potential chassis is underscored by its ability to thrive across diverse culture media and its resilience to pronounced fluctuations in light intensity and pH levels (Butler et al., 2020). Furthermore, advancements in the genetic understanding of *P. tricornutum* have catalyzed its use as a chassis for identifying novel genes and producing high-value components (Daboussi et al., 2014; De Riso et al., 2009). Given its rich biochemical profile, environmental adaptability, meticulously characterized genome, and available engineering tools, *P. tricornutum* emerges as a prospective diatom cell factory for future applications. Beyond serving as a microalgal cell factory for high-value product synthesis, *P. tricornutum* has also been established as a model organism to investigate diatom physiology in various unique environments (Jallet et al., 2016; Levitan et al., 2015; Kuczynska et al., 2020). Therefore, understanding the mechanisms of *P. tricornutum*’s response to water stress is imperative for its potential deployment in water-scarce regions.

Polyethylene glycol (PEG) compounds are utilized to induce water stress in petri dishes (*in vitro*) for plants, ensuring a consistent water potential throughout the experimental period (Sharma et al., 2022; Mahpara et al., 2022). PEG has been widely employed as an abiotic stress inducer in numerous studies aimed at screening water-stress germplasm (Peršić et al., 2022; Reyes et al., 2023). As a polymer, PEG is regarded as a superior chemical for artificially inducing water stress compared to other alternatives. The application of PEG-induced water stress effectively reduces cellular water potential (Qi et al., 2023; Reza Yousefi et al., 2020). To address this challenge, our study employed PEG as a water stress inducer to investigate the response of marine diatoms to water stress. Additionally, we elucidated the potential mechanistic relationships between the stress response and metabolic reconfiguration. These insights pave the way for leveraging marine diatom biotechnology to enhance valuable product accumulation in regions with water scarcity.

## 2 Materials and methods

### 2.1 Strain and preculture conditions

The strain *P. tricornutum* Bohlin CCMP 2561 was acquired from the Provasoli-Guillard National Center for Marine Algae and Microbiota in the United States. Cells were grown in f/2 medium, which occupies 1/3 of a 250 mL conical flask. Cultivation was maintained at 20 ± 1°C with a 12/12 h circadian rhythm and 200 μmol/m^2^/s light intensity. The cells were periodically cultured with an initial number of 10^4^ cells/mL.

### 2.2 Water stress treatments

PEG 6000 was purchased from Aladdin (Aladdin, China) as the water stress inducer. In order to explore *P. tricornutum* under water stress, various concentrations of PEG 6000 (0, 1, 3, 5, 7%, *w*/*v*) were added to the f/2 medium refer to the process in plants. Cells were harvested at specific time points for further analysis.

### 2.3 Photosynthetic parameter determination

In order to detect the activation of the photosystem, parameters such as the maximum quantum efficiency of photosystem II (Fv/Fm), non-photochemical quenching (NPQ) and chlorophyll *a* (Chl *a*), during treatments supplemented with or without PEG 6000 were determined by a commercial phytoplankton photosynthesis analyzer PhytoPAM (Walz, Germany) following the protocols retrieved from the manufacturers.

### 2.4 Primary metabolites analysis

The primary metabolites of *P. tricornutum* under water stress were determined after 3 days of exposure to PEG6000 according to a previous study (Hao et al., 2022). In brief, the total lipid content was extracted by the methanol-chloroform protocol and determined gravimetrically. The total carbohydrate was isolated by the classical phenol-sulfuric acid method and then determined by spectrophotometry at OD_562nm_. The total protein was isolated by RIPA lysis buffer (Beyotime, China) and quantified by the BCA protein assay kit (Beyotime, China).

### 2.5 Oxidant stress analysis

The ROS level of *P. tricornutum* under water stress was determined by the fluorescence probe DCFH-DA (Solarbio, China). Cells were stained with 10 μM DCFH-DA and darkly incubated at 37°C for 30 min. Then, cells were washed three times with fresh f/2 medium and the fluorescence was measured by a microplate reader (Bio-Tec, Vermont, American) with an excitation wavelength of 488 nm and an emission wavelength of 528 nm.

### 2.6 Fluorometric and fatty acid component analysis of total lipid

The content of the major lipid, neutral lipid, in *P. tricornutum* was determined every day by using Nile Red (Aladdin, China) according to a previous study (Hao et al., 2024). Fatty acid composition of total lipid was analyzed as fatty acid methyl esters (FAMEs) by using gas chromatography–mass spectrometry (GC–MS, Agilent7890B-7000C). In summary, 500 μL of toluene was introduced to 5 mg wet algae pellet and transferred into a new tube. Subsequently, 1 mL of freshly prepared 0.5 N NaOH/MeOH was added. The mixture was thoroughly vortexed and then incubated at 80°C for 20 min. Following a 5-min cooling period at room temperature, 1 mL of freshly prepared AcCl/MeOH (1:10, v/v) was gradually added, and the mixture was incubated for an additional 20 min. Next, 1 mL of 6% K₂CO_3_, 500 μL of hexane, and 10 μL of methyl nonadecylate (Aladdin, China) were incorporated, and the mixture was vortexed for 1 min. The upper phase was subsequently collected for GC–MS analysis. A DB-5 quartz capillary column measuring 30 m × 0.25 mm × 0.25 μm was employed for chromatographic separation. The column temperature program began with an initial hold at 60°C for 1 min, followed by a ramp at 10°C per minute to 160°C, and then a gradual increase at 2.5°C per minute to a final temperature of 250°C. The injector was maintained at 280°C, and 1 μL of sample was injected in splitless mode. The mass spectrometer’s transfer line was set to 200°C. Fatty acids were identified using the NBS spectral library integrated into the system, and quantification was performed by analyzing the integrated peak areas.

### 2.7 Statistical analysis

All experiments were performed in at least biological triplicates to ensure reproducibility. GraphPad Prism 8.0 software was employed to analyze the data, which were expressed as mean ± standard deviation (SD) (*n* = 3).

## 3 Results

### 3.1 Effects of water stress on growth of *Phaeodactylum tricornutum*

**Figure 1:**
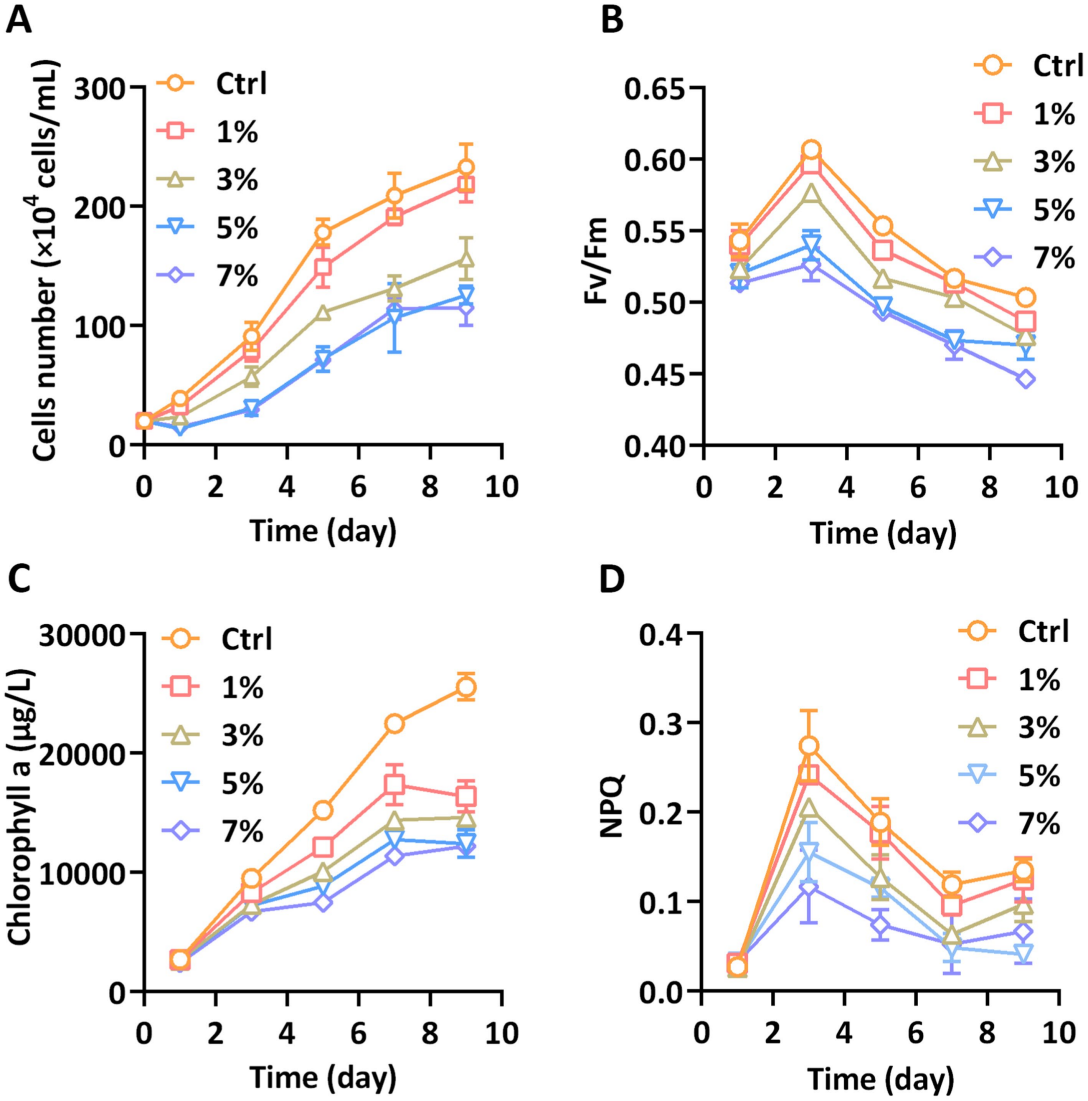
Growth and photosynthesis parameters **(A)** Cells number, **(B)** Fv/Fm, **(C)** Chlorophyll a, and **(D)** NPQ.

As shown in Figure 1A, the lower concentration of PEG of 1% had no significant effect on growth rate of *P. tricornutum* during the cultivation period. At a PEG concentration of 3%, the growth rate of cells was significantly repressed compared to that in the control group. The cell density declined after 1 day with the addition of 3% PEG. While the concentration of PEG was increased to 5%, the growth rate decreased more than 3%, even more than the initial cell density. The inhibitory effect of 7% PEG was similar to that of 5% and the growth rate had no further decrease. Corresponding to the growth rate, the Fv/Fm values were lower for *P. tricornutum* above 3% PEG compared to the control. As shown in Figure 1B, there was a trend toward lower Fv/Fm of 5 and 7% PEG at day 1, and this trend was continued during the cultivation period. Furthermore, the NPQ value, which indicated the dissipation capacity of excessive light energy, was also repressed above 3% PEG (Figure 1C). The content of chlorophyll a directly represents the light-harvested photosynthesis of diatoms. The biosynthesis of chlorophyll a was significantly blocked under all treatments of PEG (Figure 1D). These analyses showed that water stress induced by PEG impairs the growth and photosynthetic activity of *P. tricornutum*.

### 3.2 Antioxidant analysis of *Phaeodactylum tricornutum*

**Figure 2:**
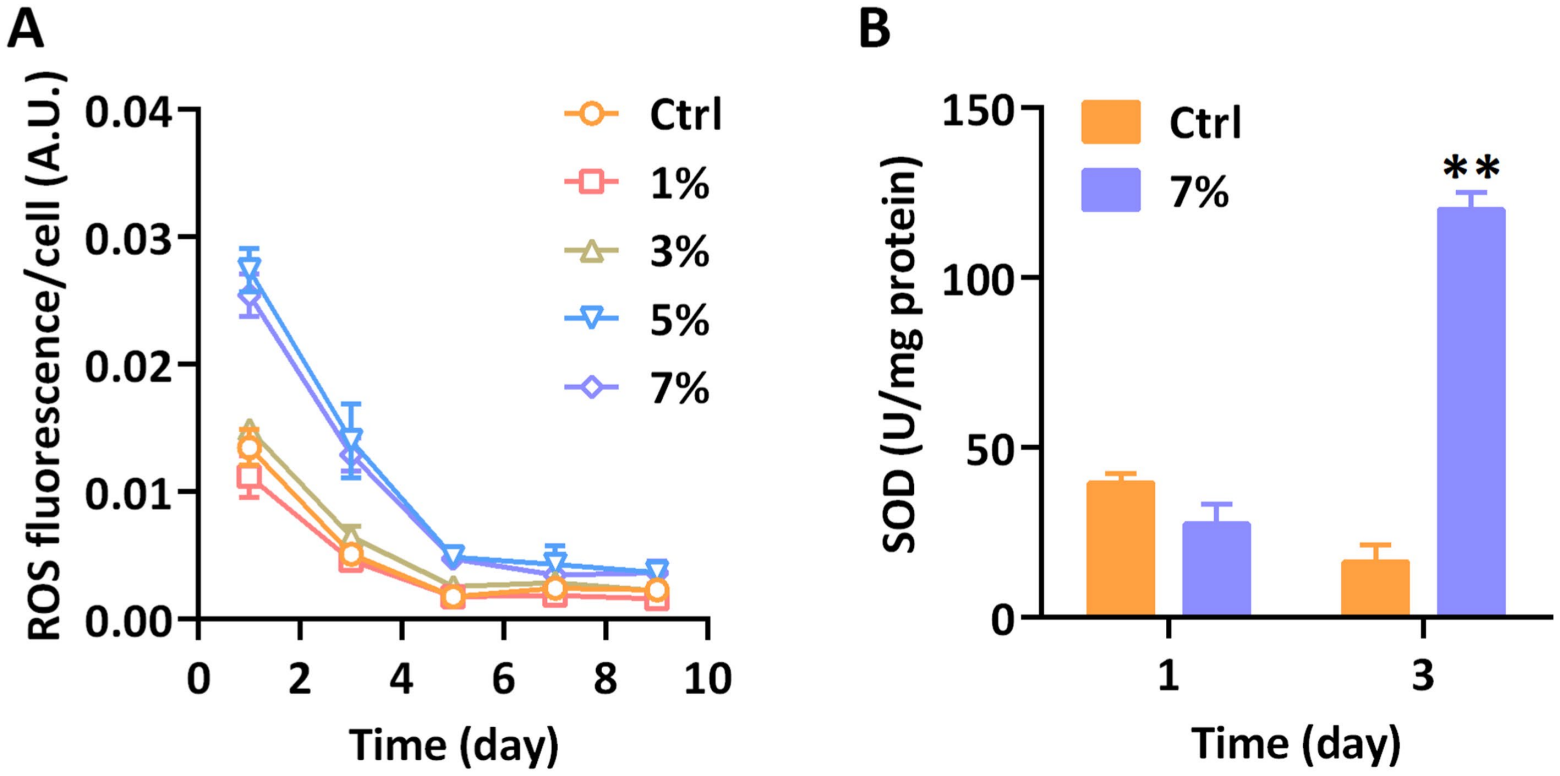
The antioxidant capacity of *P. tricornutum* exposed to water stress. **(A)** ROS fluorescence and **(B)** SOD activity. Significant difference is indicated at *p* < 0.05 (*) or *p* < 0.01 (**) level. Each value represents mean ± SD (*n* = 3).

We tracked and monitored the ROS level of *P. tricornutum* treated with PEG to evaluate the tolerance under water stress. As shown in Figure 2A, the water stress treatment with highly concentrated PEG significantly induced the release of ROS in *P. tricornutum*. With the monitoring of DCFH-DA, the fluctuating of ROS fluorescence had not been observed under the action of 1 and 3% PEG, however, the ROS levels per cell under 5 and 7% PEG were similar and significantly higher than those in the control group from the 1st to the 5th day. Along with the prolongation of the culture time, the ROS fluorescence strength of all processing groups had been consistent after 5^th^ day. Our results showed that 5 and 7% PEG triggered a significant accumulation of ROS in *P. tricornutum*. Superoxide dismutase (SOD) is directly involved in the formation of ROS, whose activity is considered a critical indicator of oxidative stress. In order to further investigate the reasons for the significant increase in ROS content on days 1 and 3, we measured the SOD enzyme activity of *P. tricornutum*. As shown in Figure 2B, the SOD activity had no significant effect at the earlier stage of PEG cultivation, but followed a remarkable increase at day 3, showing a hysteretic reaction after the release of ROS. Our results presented the water stress, induced oxidative stress of *P. tricornutum*.

### 3.3 Determination of TAG content

**Figure 3:**
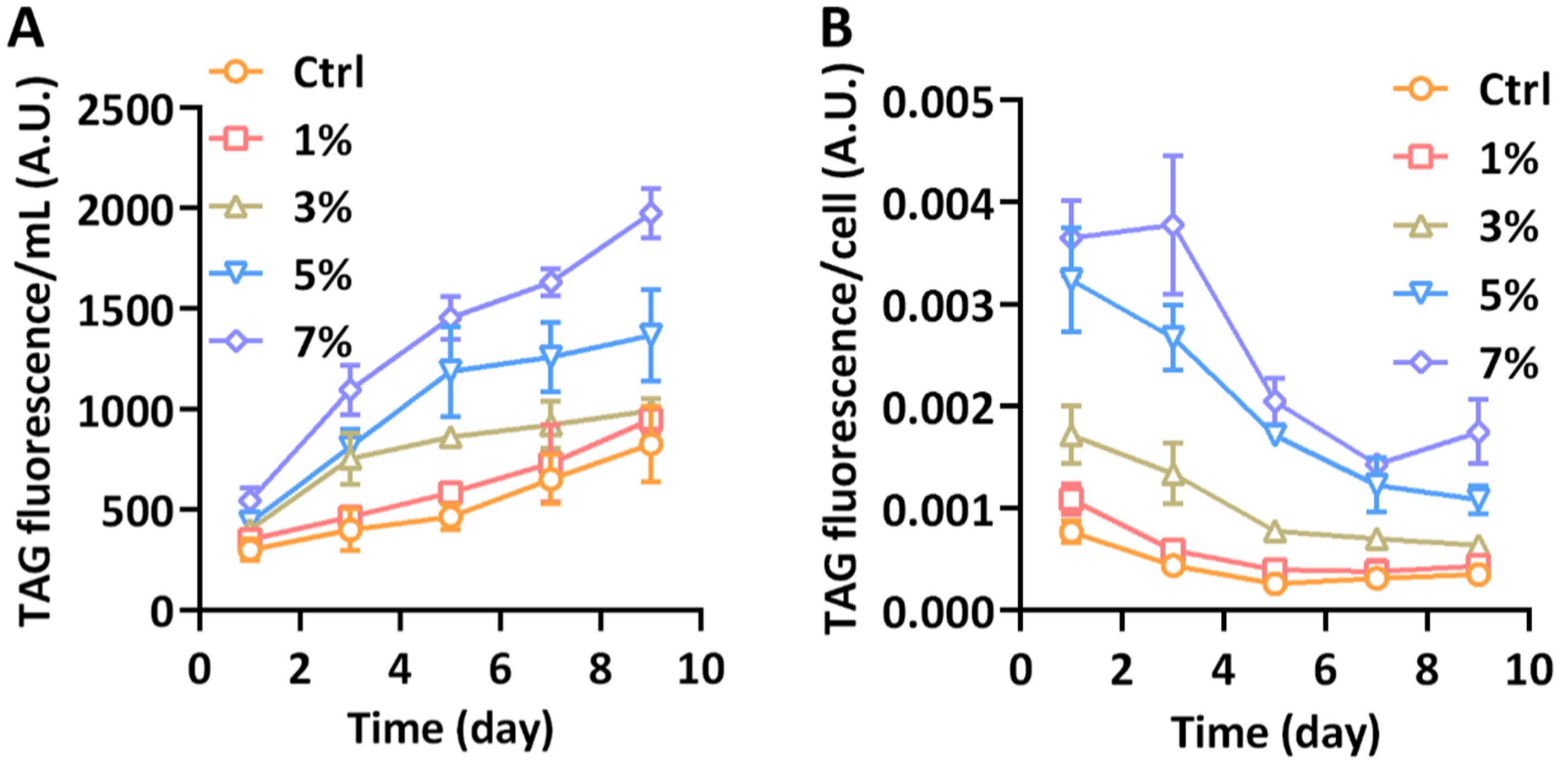
TAG content measured with Nile Red fluorometric analysis. **(A)** The total TAG fluorescence per milliliter; **(B)** The TAG fluorescence per cell.

As the iconic lipid in *P. tricornutum*, triacylglycerol (TAG) could be accumulated when cells were immersed in abiotic stresses. We employed the fluorescence intensity of Nile Red to monitor the accumulation of TAG. The quantitative fluorescence of Nile Red combined with TAG is shown in Figure 3A. Compared with the control group, 1% PEG had no significant effect on the TAG content of *P. tricornutum*. Significant accumulation of TAG was presented when the PEG concentration reached 3% and was further induced under 5 and 7% PEG. The total content of TAG in the equal volume of medium was richest under 7% PEG concentration on the 9th day. Considering the opposite effects of increased total fluorescence and declining cell numbers, the content of TAG per cell was further detected. As shown in Figure 3B, the content of TAG was still significantly increased under 7% PEG; however, it declined after day 3 due to the prolonged cultivation. Our results implied that water stress could induce a significant accumulation of TAG in *P. tricornutum*.

### 3.4 Determination of primary metabolites and fatty acids

**Figure 4:**
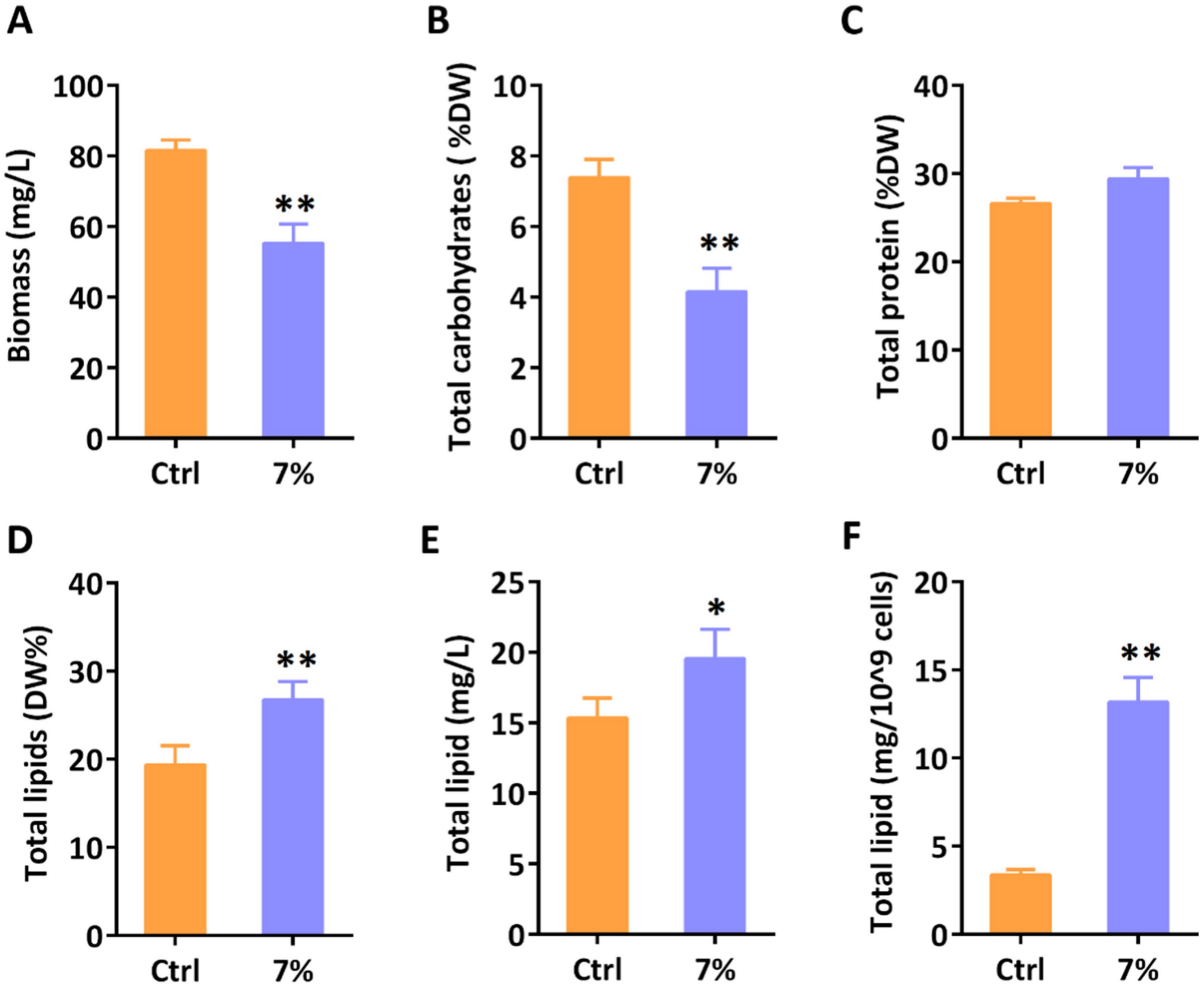
Determination of primary metabolites under water stress on day 3. **(A)** Total biomass; **(B)** Total carbohydrates content; **(C)** Total protein content; **(D)** Total lipids content; **(E)** Total lipids content per liter; **(F)** Total lipids content per 10^9^ cells. Significant difference is indicated at *p* < 0.05 (*) or *p* < 0.01 (**) level. Each value represents mean ± SD (*n* = 3).

Water stresses are known to influence the redistribution of primary metabolites in various systems. As the major primary component in *P. tricornutum*, the variation in total lipid content indicated the redistribution of primary metabolites. Hence, we determined the primary metabolites of *P. tricornutum* under 7% PEG treatment by biochemistry and gravimetric analysis. As shown in Figure 4A, the gravimetric analysis revealed that the total biomass of microalgae was significantly decreased under water stress, which was coincided with the decrease in cell number. Furthermore, we determined the three major primary metabolites in *P. tricornutum* to explore the distribution of energy. Biochemistry analysis revealed that the content of total proteins was kept in balance with the control group, however, the total lipid content was elevated along with the reduced carbohydrates (Figures 4B–D). In industrial application scenarios, the total production of metabolites and their proportion to cell dry weight often exhibit different trends. Then, we determined the total lipid content on a large scale (Figures 4E, F). Conventional gravimetric analysis revealed that the total lipid content was found to increase remarkably when 10^9^ cells and 1 L of algae were used as units of measurement, respectively. Our results indicated that water stress stimulates metabolic flow toward lipid synthesis.

**Figure 5:**
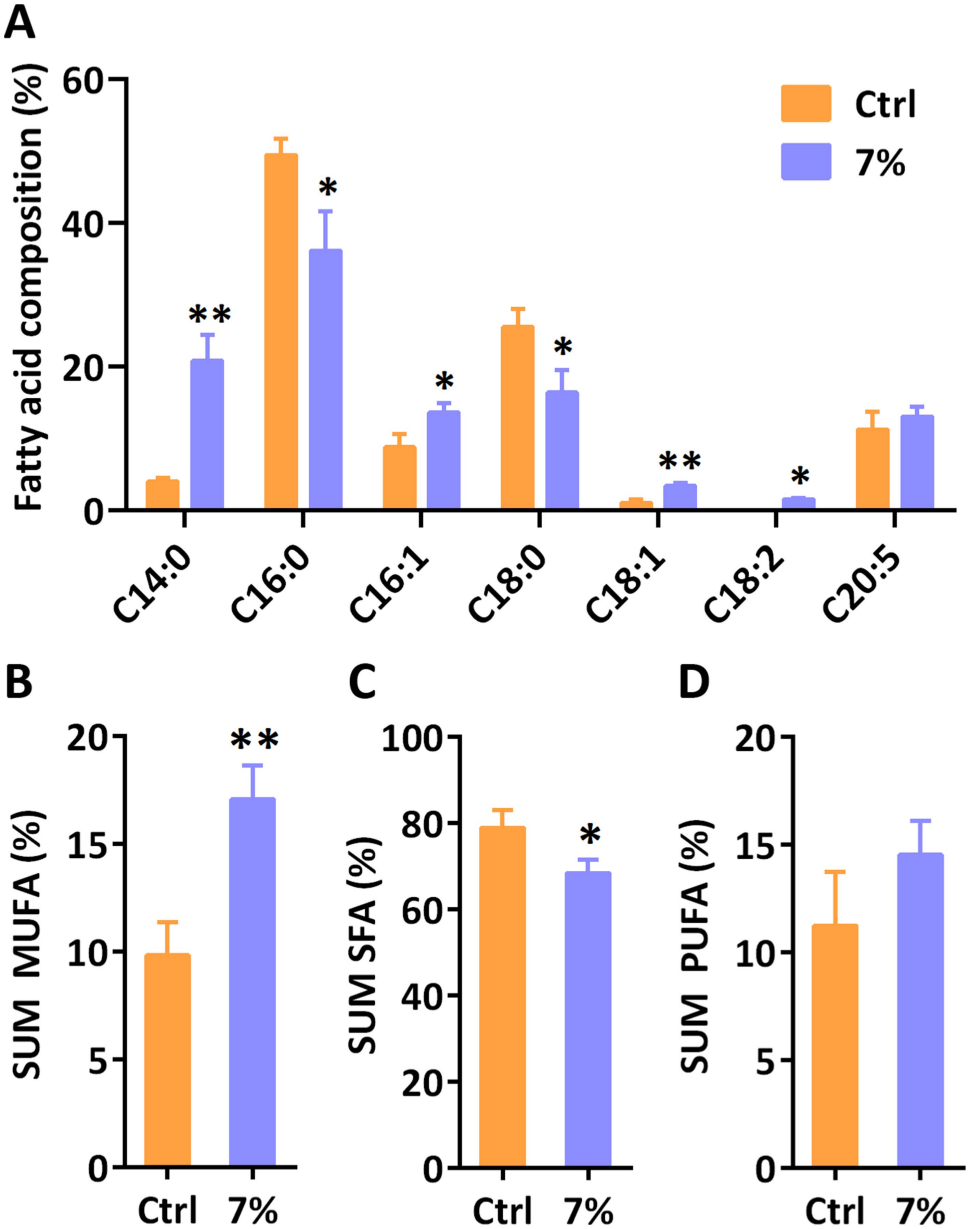
The relative fatty acid content **(A)** and sum monounsaturated fatty acids (MUFA) **(B)**, saturated fatty acids (SFA) and polyunsaturated fatty acids (PUFA) in *P. tricornutum* under water stress induced by 7% PEG. Significant differences are indicated at *p* < 0.05 (*) or *p* < 0.01 (**) level. Each value represents mean ± SD (*n* = 3).

Furthermore, we analyzed the relative fatty acid composition of total lipid at day 3 under 7% PEG. As shown in Figure 5, the content of monounsaturated fatty acids (MUFA) in total fatty acids increased while the content of saturated fatty acids (SFA) decreased. Among them, the proportions of C14:0, C16:1 and C18:1 were increased, while C16:0 and C18:0 were decreased under water stress compared to the control group, respectively. However, the accumulation of one of the most valuable polyunsaturated fatty acids (PUFA), eicosapentaenoic acid (EPA, C20:5), in *P. tricornutum* was not disturbed under water stress. These results demonstrated that water stress is involved in the *de novo* synthesis of fatty acids in *P. tricornutum*, especially regulating MUFA and SFA.

## 4 Discussion

Bio-products derived from diatoms are increasingly viewed as sustainable alternatives to traditional chemical products in both the fuel and pharmaceutical sectors (Wang and Seibert, 2017). However, the large-scale cultivation of diatoms presents significant economic and water resource challenges, which hinder the exploitation of microalgae resources in regions with limited water availability (Piano et al., 2017; Jager et al., 2019; Arenas-Sánchez et al., 2016). While numerous studies have examined the mechanisms of water stress in higher plants and proposed strategies to address water scarcity (Farooq et al., 2012; Basu et al., 2016; Fathi and Tari, 2016), there remains a dearth of research on the mechanisms diatoms employ under water stress. This underscores the imperative to investigate the physiology and metabolism of diatoms in such conditions.

In this study, PEG 6000 was utilized to simulate water stress in the cultivation of *P. tricornutum* CCMP2561, as a model organism for studying the physiological response of diatoms under various abiotic stressors. Our results highlighted the pronounced impact of water stress on photosynthetic growth and metabolism of *P. tricornutum*, which will expand the physiological mechanism of water stress on other diatoms and microalgae. Previous studies have documented the disruptive effects of water stress on photosynthetic systems (Reddy et al., 2004; Wang et al., 2018). The reducing Fv/Fm and NPQ implied the damages in photosynthetic organs under water stress. Abiotic stress can lead to a weakness in photosynthetic capacity of chloroplast, which release signal to change the energy balance for adapting stress (Woodson, 2022; Li and Kim, 2022). Our data indicate that when exposed to levels above 3% PEG, *P. tricornutum* exhibited reductions in growth rate, photosynthetic efficiency, and chlorophyll a content. These findings align with prior observations in terrestrial plants, suggesting analogous photosynthetic responses to water stress in both microalgae and plants. Evolutionary biology establishes that plant chloroplasts share homology with microalgae (McFadden, 2001), further pointing to a shared vulnerability in chloroplasts to water stress-induced damage affecting light harvesting and cell division.

Water stress is known to induce oxidative stress, adversely affecting the physiology and metabolism of higher plants (Impa et al., 2012; Kaur and Asthir, 2017). In our study, we noted a significant release of ROS in *P. tricornutum* under water stress, aligning with prior observations in plants. This suggests a consistent oxidative stress response in both diatoms and higher plants when exposed to water stress. Interestingly, we observed that ROS content in microalgae peaked on the first day of PEG treatment, regardless of the hydration status of the microalgae, and subsequently decreased over the course of cultivation. A comparable trend was identified in *Auxenochlorella pyrenoidosa* during its bioremediation of hazardous wastewater (Wang et al., 2022). Such findings hint at a potential adaptive mechanism in microalgal cells to navigate novel environments. However, the SOD enzyme activity, crucial in neutralizing free radicals and thus alleviating stress in microalgae (Aderemi et al., 2018; Gauthier et al., 2020), did not display an immediate surge alongside the ROS increase on the first day. This lag in SOD activity elevation post-excessive ROS release might be attributed to the temporal disconnect between signal transduction and enzyme expression.

Metabolic redistribution under abiotic stress significantly influences the lipogenesis in diatoms. While numerous studies have highlighted the augmented accumulation of lipids in diatoms due to various abiotic stressors, such as excess light or nutrient deprivation (e.g., nitrogen or phosphorus starvation) (Levitan et al., 2015; Yang et al., 2014; Huete-Ortega et al., 2018), lipogenesis metabolism under water stress remains unexplored. Notably, diatoms often adapt by curtailing their growth rate and redirecting metabolic pathways toward lipogenesis during abiotic stress, ensuring cellular viability. Our study revealed the plasticity of diatoms in accumulating triglycerides under water stress. However, the weakened photosynthetic autotrophic ability hinders the accumulation of biomass under diatom water stress. The mixotrophic cultivation based on adding external organic sources can compensate for the lack of diatom biomass (Hao et al., 2020). Our study used PEG as an inducer to investigate the response of diatoms to water stress. However, PEG cannot reveal the real water stress conditions for maintaining the suspension ability of diatom cells in liquid environment. In the future, we will build a more reasonable water stress model and explore in depth the stress response mechanism of diatoms based on the results of this study. In addition, further exploration of the molecular mechanisms of diatom under water stress will be beneficial for screening water stress resistant genes to construct stress resistant algal strains using synthetic biology. Consistent with this, our findings indicate an increased lipid content under water stress, underscoring the parallels in physiological responses of diatoms and higher plants to water stress.

## 5 Conclusion

This study investigated the physiological and metabolic responses of *P. tricornutum* to water stress. We observed a significant induction of ROS release, inhibiting photosynthetic growth. Furthermore, water stress led to a notable increase in total lipid content and altered the fatty acid composition. Together, these findings offer insights into the potential utilization of diatoms in areas with water scarcity.

## Data Availability

The raw data supporting the conclusions of this article will be made available by the authors, without undue reservation.
